# Presurgical Localization of the Primary Sensorimotor Cortex in Gliomas: When is Resting State FMRI Beneficial and Sufficient?

**DOI:** 10.1007/s00062-020-00879-1

**Published:** 2020-04-09

**Authors:** Natalie L. Voets, Puneet Plaha, Oiwi Parker Jones, Pieter Pretorius, Andreas Bartsch

**Affiliations:** 1 Wellcome Centre for Integrative Neuroimaging, FMRIB Centre, John Radcliffe Hospital, University of Oxford, OX3 9DU Headington, Oxford, UK; 2 Department of Neurosurgery, John Radcliffe Hospital, Oxford University Hospitals NHS Foundation Trust, Oxford, UK; 3 Department of Neuroradiology, John Radcliffe Hospital, Oxford University Hospitals NHS Foundation Trust, Oxford, UK; 4 Department of Neuroradiology, University of Heidelberg, Heidelberg, Germany

**Keywords:** Tumor, Task fMRI, ICA, Sensorimotor, Surgery

## Abstract

**Purpose:**

Functional magnetic resonance imaging (fMRI) has an established role in neurosurgical planning; however, ambiguity surrounds the comparative value of resting and task-based fMRI relative to anatomical localization of the sensorimotor cortex. This study was carried out to determine: 1) how often fMRI adds to prediction of motor risks beyond expert neuroradiological review, 2) success rates of presurgical resting and task-based sensorimotor mapping, and 3) the impact of accelerated resting fMRI acquisitions on network detectability.

**Methods:**

Data were collected at 2 centers from 71 patients with a primary brain tumor (31 women; mean age 41.9 ± 13.9 years) and 14 healthy individuals (6 women; mean age 37.9 ± 12.7 years). Preoperative 3T MRI included anatomical scans and resting fMRI using unaccelerated (TR = 3.5 s), intermediate (TR = 1.56 s) or high temporal resolution (TR = 0.72 s) sequences. Task fMRI finger tapping data were acquired in 45 patients. Group differences in fMRI reproducibility, spatial overlap and success frequencies were assessed with t‑tests and χ^2^-tests.

**Results:**

Radiological review identified the central sulcus in 98.6% (70/71) patients. Task-fMRI succeeded in 100% (45/45). Resting fMRI failed to identify a sensorimotor network in up to 10 patients; it succeeded in 97.9% (47/48) of accelerated fMRIs, compared to only 60.9% (14/23) of unaccelerated fMRIs ($\chi$(2) = 17.84, *p* < 0.001). Of the patients 12 experienced postoperative deterioration, largely predicted by anatomical proximity to the central sulcus.

**Conclusion:**

The use of fMRI in patients with residual or intact presurgical motor function added value to uncertain anatomical localization in just a single peri-Rolandic glioma case. Resting fMRI showed high correspondence to task localization when acquired with accelerated sequences but offered limited success at standard acquisitions.

**Electronic supplementary material:**

The online version of this article (10.1007/s00062-020-00879-1) contains supplementary material, which is available to authorized users.

## Introduction

Functional magnetic resonance imaging (fMRI) is widely used to inform and guide neurosurgery for intra-axial lesions and epileptogenic foci. Surprisingly, there remains little consensus around when fMRI is most useful and how functions should be mapped.

Neurological risks are particularly high around the precentral gyrus [1] due to absolute cortical representation of primary sensorimotor functions [2]. Nevertheless, substantial interindividual variability exists in cytoarchitecture [3] and in cortical specialization along the central sulcus [4]. Difficulties in predicting function from anatomy, coupled with the potential for pathological distortion, motivated motor mapping as the first clinical application of fMRI. Subsequently, controversy has developed around fMRI’s usefulness to improve neurosurgical planning. Some groups [5] proposed that task fMRI (tfMRI) improves delineation of the hand motor area relative to anatomical landmarks in patients with mass lesions. Others note only limited added value [6].

Success of tfMRI depends on patients’ compliance, existing motor deficits, and confounding effects such as head movement. Additionally, multiple tasks are needed to map different subregions along the motor homunculus [7]. Resting state fMRI (rsfMRI) has been proposed as an alternative to identify the sensorimotor network without active patient participation and it has demonstrated potential to localize primary motor and sensory regions equally well as tfMRI [8, 9], including in brain tumor patients [10, 11]. Conversely, typical data-driven resting-derived sensorimotor networks offer no specificity to separate functional subregions along the central sulcus.

Recent developments in accelerated image acquisition techniques may improve the specificity of clinical rsfMRI scans. In healthy volunteers scanned for the Human Connectome Project, high temporal resolution rsfMRI provided separation between the hand, foot and mouth motor regions [12]. The extent to which these advantages might support presurgical planning and intraoperative neuronavigation is not yet known.

This study compared anatomical and functional MRI data acquired from healthy controls and patients with a tumor either affecting or distal to the central region. It was hypothesized that expert radiological review predicts functional risks in most if not all patients. Furthermore, among fMRI approaches, it was predicted that accelerated rsfMRI would provide comparable sensitivity to tfMRI to localize the motor strip in surgical candidates. To this aim it was tested: 1) how often fMRI adds to the prediction of motor-related surgical risks, beyond anatomical localization of the central sulcus, 2) the success rate of rsfMRI and tfMRI sensorimotor mapping, and 3) the impact of temporal sampling rate on network detectability by rsfMRI.

## Material and Methods

### Participants

Data from 71 patients (31 women) with an intra-axial primary brain tumor recruited prospectively between January 2014 and June 2018 were retrospectively reviewed. Patients were recruited prior to planned neurosurgery following review by a multidisciplinary neuro-oncology team. Patients came from two sites. Additionally, 14 healthy volunteers (6 women) were recruited at 1 site for normative and test-retest comparisons. Inclusion criteria were: aged 18 years or above, normal or corrected to normal vision. Exclusion criteria were: contraindications to MRI, prior resections and radiotherapy/chemotherapy. All participants gave prospective informed written consent to take part in the study. The study was approved by the South Central-Oxford B Research Ethics Committee in Oxford and the Local Ethics Committee in Heidelberg (based on a previous approval by the Local Ethics Committee of the University of Würzburg).

The surgical strategy was established for all patients according to a structured protocol for clinical decision making, which was tailored on a case by case basis according to tumor location and extent (as well as potential multifocality and the presence of subependymal or leptomeningeal spread, if applicable). In general, patients were primarily evaluated on an outpatient or consultation basis by a consultant neurosurgeon, who reviewed the available clinical as well as imaging information and examined the patient neurologically and with respect to performance/compliance status. At this point, all treatment options including no surgery, biopsy only, debulking/tumor reduction or gross total resection were considered. Predicated on this initial evaluation, each patient was then reviewed in a multidisciplinary neurosurgical/neuro-oncological tumor board. Based on this joint case discussion, informed by any supplementary imaging when meanwhile obtained, the patient’s likely tolerance of intraoperative monitoring (e.g. electrophysiology and/or awake surgery) and if relevant, adjuvant treatment options, the surgical plan was then ratified or amended. The final plan was communicated with and decided on informed consent of the patient. Importantly, fMRI results were used to inform but not to decide the surgical strategy; indications for intraoperative monitoring (motor evoked potentials [MEP], somatosensory evoked potentials [SEP] or spinal somatosensory evoked potentials [SSEP] phase reversal and/or awake surgery with cortical/subcortical stimulation mapping) according to standardized protocols were always based on proximity to cortical/subcortical structures identified anatomically.

### Magnetic Resonance Imaging

Patients were scanned on one of three Siemens 3T MRI (Siemens Healthineers, Erlangen, Germany) systems; a Verio (*n* = 23), Trio (*n* = 22) or Prisma (*n* = 26) scanner. Healthy volunteers (*n* = 14) were scanned twice on the Prisma, 6 months apart (online resource methods).

Scans consisted of a 1 mm^3^ T1-weighted MPRAGE (Magnetization Prepared RApid Gradient Echo) scan, followed by blood oxygen-level dependent gradient-echo echo-planar imaging (EPI) rsfMRI covering the entire brain. The rsfMRI data were collected once per patient, either at standard unaccelerated (TR = 3.5 s), intermediate (TR = 1.56 s) or fast (TR = 0.72 s) temporal sampling (Table 1). For the latter data acquired by simultaneous multislice acquisitions (Trio/Prisma scans), a reference volume providing higher tissue contrast was collected to facilitate registration of each individual’s functional to structural images. For rsfMRI, participants were requested to lie still and rest while watching a fixation cross. The tfMRI data were acquired after rsfMRI in 45 patients and all 14 controls. Dominant hand knob activations were evaluated using motor trials implemented in a fMRI adaptation of the Corsi block tapping test (online resource Fig. S1).

**Table 1:** fMRI sequence acquisition parameters

System	Resting fMRI	Task fMRI
Verio	Sample: *n* = 23 patients. standard sequence: TR = 3.5 s, TE = 30 ms, flip angle = 90°, voxel resolution = 2 × 2 × 2 mm, 54 slices providing whole-brain coverage, 85 volumes, duration: 05:10 min	Sample: *n* = 21 patients. TR = 3.0 s, TE = 28 ms, flip angle = 90°, voxel resolution = 3 × 3 × 3 mm, 44 slices providing whole-brain coverage. total number of motor trials = 112, duration 56 s (among full task duration of 07:30 min)
Prisma	Sample: *n* = 26 patients, *n* = 14 controls. Ultra-high temporal resolution accelerated sequence: TR = 0.72 s, TE = 32 ms, flip angle = 50°, multiband acceleration factor = 8, voxel resolution = 2 × 2 × 2 mm, 72 slices providing whole-brain coverage, 500 volumes, duration: 6:09 min	Sample: *n* = 24 patients, *n* = 14 controls. TR = 0.93 s, TE = 33.4 ms, multiband acceleration factor = 6, flip angle = 64°, voxel resolution = 2 × 2 × 2 mm, 72 slices providing whole-brain coverage
Trio	Sample: *n* = 22 patients. Intermediate temporal resolution accelerated sequence: TR = 1.56 s, TE = 30 ms, flip angle = 70 °, multiband acceleration factor = 3, 54 slices providing whole-brain coverage at 1.8 × 1.8 × 2.25 mm voxel resolution, 250 volumes, duration: 06.50 min	Not acquired

Acquisition parameters for three 3 T Siemens MRI scanners

Generally, one resting fMRI dataset was acquired for every patient, due to time constraints for presurgical planning in patients with, at least in part, limited compliance and scanning tolerance. In one patient, however, rsfMRI was repeated to directly compare basic and intermediate acceleration sequences for the same individual, see online resource Fig. S4, to illustrate the advantage of increased temporal resolution on a voxel-wise/vertex-wise basis within subject

*TR* repetition time, *TE* echo time

### Central Sulcus (CS) Identification

**Fig. 1:**
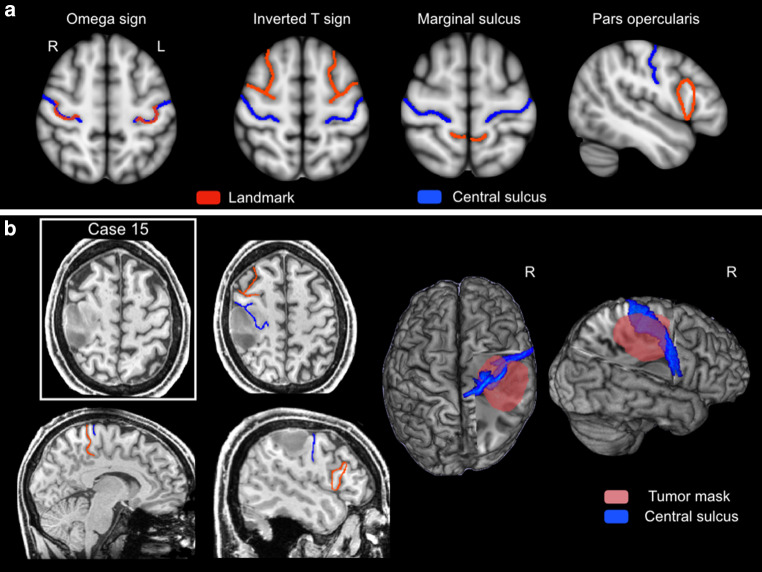
Anatomical landmarks identifying the central sulcus. Two consultant neuroradiologists independently identified the central sulci (CS) in the 71 participants’ T1-weighted anatomical scan. **a** Template MNI152 brain illustrating the combination of four well-established landmarks (*red lines*) used to locate the CS (*blue line*): the inverted omega which can be shallow or doubled (see Fig. 3); the inverted T sign; the “pli de passage fronto-pariétal supérieur”; and the termination of precentral gyrus behind pars opercularis. The lowest extension of the precentral gyrus posteriorly turns into the subcentral gyrus (not marked), which is also helpful for orientation. **b** Application of these landmarks to localize the central sulcus in a patient (Case 15 in Table 4) with severe effacement of the CS due to tumor infiltration and mass effect. Here, the location of the CS (*blue line*) was not unambiguous but determined on consensus re-review to course through the body of the tumor (outlined in red on the 3D brain rendering)

The CS was localized in each hemisphere on every individual’s anatomical scan by two experienced (>15 years) board-certified neuroradiologists working independently and blinded to fMRI results. It was identified using four landmarks: (i) inverted omega of the precentral gyrus [13], (ii) the inverted T sign at the termination of the superior frontal sulcus at the precentral gyrus [14], (iii) marginal ramus of the cingular sulcus or „pli de passage fronto-parietal superieur“ [15] and (iv) termination of precentral gyrus behind the pars opercularis ([16]; Fig. 1). The lowest extension of the precentral gyrus posteriorly turns into the subcentral gyrus, or „pli de passage fronto-parietal inferieur“, which can also be helpful for orientation. Rarely, the pre- and postcentral gyrus are connected by a third „pli de passage fronto-parietal moyen“ of Broca at the level of the hand knob.

### fMRI Analyses

The fMRI data were analyzed using the FMRIB Software Library (FSL) [17]. Preprocessing consisted of brain extraction, high pass filtering (cut-off at 90 s), spatial smoothing at 5 mm (full width at half maximum), distortion correction using field maps, and affine registration to structurals using boundary-based registration. Spatiotemporal components were automatically estimated from each individual’s data using independent component analysis (ICA) [18].

For tfMRI, post hoc regression was used to identify ICA components where temporal signal fluctuations correlated with task trial timings (modeled using gamma convolved hemodynamic response functions) [18, 19]. Resulting task ICA components were thresholded using default Gaussian gamma mixture modeling (*p* > 0.5, denoting a higher probability of reflecting true signal than noise).

In rsfMRI, individual (sensori)motor networks were identified in two ways: first, spatial maps from the single subject resting ICA were inspected to identify a characteristic network overlapping the precentral and postcentral gyri. Visual inspection is practical in clinical settings, but subjective. Therefore, a second automated dual regression (DR) approach [20] was also performed, which objectively extracts spatial components in individual datasets that match given template resting state networks (RSNs). Each subject’s rsfMRI data were regressed against 10 extensively validated RSNs [8]. From the resulting spatial maps, the sensorimotor network was selected and Gaussian Gamma mixture model thresholding (*p* > 0.5) was applied to match the task analysis.

### Localization to M1

For tfMRI and rsfMRI, the extent of spatial overlap between fMRI maps and the probabilistic location of the primary motor cortex (M1) were determined based on the Jülich histological template atlas [21]. Overlap between fMRI and M1 was measured based on a voxel count and Dice similarity metrics (online resource methods).

### Generalizability Analysis

The impact of temporal fMRI resolution was evaluated by statistically comparing frequencies of sensorimotor RSN detections according to temporal sampling rate, and the number of functional subdivisions identified from single-subject resting ICAs between scan groups.

### Pathological Confounds

Potentially confounding effects of tumor grade and location were evaluated by comparing fMRI network detection rates according to tumor location (distal/affecting the central region) and histopathology (World Health Organization grading).

### Motor Assessment

Muscle strength was evaluated using the Medical Research Council (MRC) scale before surgery, during hospitalization and up to 3 months after surgery (Table 2).

**Table 2:** Motor performance in patients before and following surgery

Group	Presurgery	0–48 h postsurgery	3 months postsurgery
All patients (*n* = 71)	Intact: *n* = 66/71 Weak: *n* = 5/71	Intact: *n* = 56/68 Weak: *n* = 12/68	Intact: *n* = 64/68 Weak: *n* = 4/68
Tumor involving or displacing the peri-Rolandic cortex (*n* = 34)	Intact: *n* = 29/34 Weak: *n* = 5/34	Intact: *n* = 22/34 Weak: *n* = 12/34	Intact: *n* = 30/34 Weak: *n* = 4/34
Tumor not affecting M1 (*n* = 37)	Intact: *n* = 37/37 Weak: *n* = 0/37	Intact: *n* = 37/37 Weak: *n* = 0/37	Intact: *n* = 37/37 Weak: *n* = 0/37

Baseline and postsurgical muscle strength in brain tumor patients, scored using the Medical Research Council scale. Intact performance (score 5/5) and weakness (score 0–4) were rated for upper and lower limbs prior to surgery, immediately postsurgery and at clinical follow-up (3 months after surgery)

### Statistical Analyses

Statistical analyses were performed using SPSS v25 (IBM Statistics SPSS Version 25; IBM Corp., Armonk, NY, USA; [22]). Assumptions around equality of variance were verified using Levene’s test within SPSS. Paired t‑tests were used to assess correspondence between resting and task-derived measures within groups and reproducibility among controls. Independent sample t‑tests were used to compare clinical and imaging metrics between participant subgroups and χ^2^-tests were used to compare frequencies of successful CS identifications by tfMRI and rsfMRI according to temporal sampling or clinical variables (tumor location, histology). Statistical significance was set at *p* < 0.05.

## Results

### Participants

Age did not differ between patients (mean 41.9 ± 13.9 years) and controls (37.9 ± 12.7 years, t = −1.05, *p* = 0.31). Clinical data are reported in Table 3. Glioma diagnosis was histologically confirmed in 70/71 patients (98.6%), 1 patient refused a biopsy. In 34/71 (47.9%) patients, the tumor directly involved the precentral or postcentral gyrus (*n* = 23) or displaced/distorted the CS (*n* = 11).

**Table 3:** Demographic and clinical data

Group	Age (years) (mean, SD, range)	Gender (M:F)	Handedness (R:L)	Tumor location	WHO tumor grade	Histological type
Healthy controls (*n* = 14)	37.9 (12.7, 27–68)	(8:6)	(13:1)	–	–	–
Tumor patients Verio (*n* = 23)	37.2 (12.7, 20–56)	(16:7)	(20:3)	LFL: 6 LTL: 5 L Ins: 2 RFL: 4 RTL: 4 R Ins: 2	I: 1 II: 9 III: 10 IV: 2 N/A: 1a	Astrocytoma: 14 Oligodendroglioma: 5 Glioblastoma: 2 DNET:1 N/A: 1^a^
Tumor patients Prisma (*n* = 26)	42.2 (13.3, 19–70)	(13:13)	(24:2)	LFL: 9 LTL: 4 L Ins: 3 RFL: 2 RTL: 3 R Ins: 5	I: 0 II: 14 III: 8 IV: 4	Astrocytoma: 15 Oligodendroglioma: 5 Glioblastoma: 5 Diffuse glioma NOS: 1
Tumor patients Trio (*n* = 22)	46.4 (14.7, 27–69)	(11:11)	(20:2)	LFL: 9 LTL: 4 L Ins: 1 L PL: 2 RFL: 3 RTL: 0 R Ins: 2 R PL:1	I: 0 II: 11 III: 7 IV: 4	Astrocytoma: 12 Oligodendroglioma: 5 Glioblastoma: 4 Gliomatosis: 1

*M* male, *F* female, *DNET* dysembryoplastic neuroepithelial tumor, *NOS* not otherwise specified, *L* left, *R* right, *FL* frontal lobe, *TL* temporal lobe, *PL* parietal lobe, *Ins* insula, *WHO* World Health Organization

^a^One patient initially refused all surgical treatment including biopsy, opting for watch-and-wait. This patient later showed radiological transformation, at which time biopsy confirmed an anaplastic astrocytoma

### CS Identification

Anatomical CS localization was consistent between raters in 70/71 patients (98.6%). In one patient, severe effacement due to tumor infiltration confounded CS identification (Fig. 1b). In this case, CS was localized by consensus on re-review of landmark criteria.

### fMRI Success Rates

**Fig. 2:**
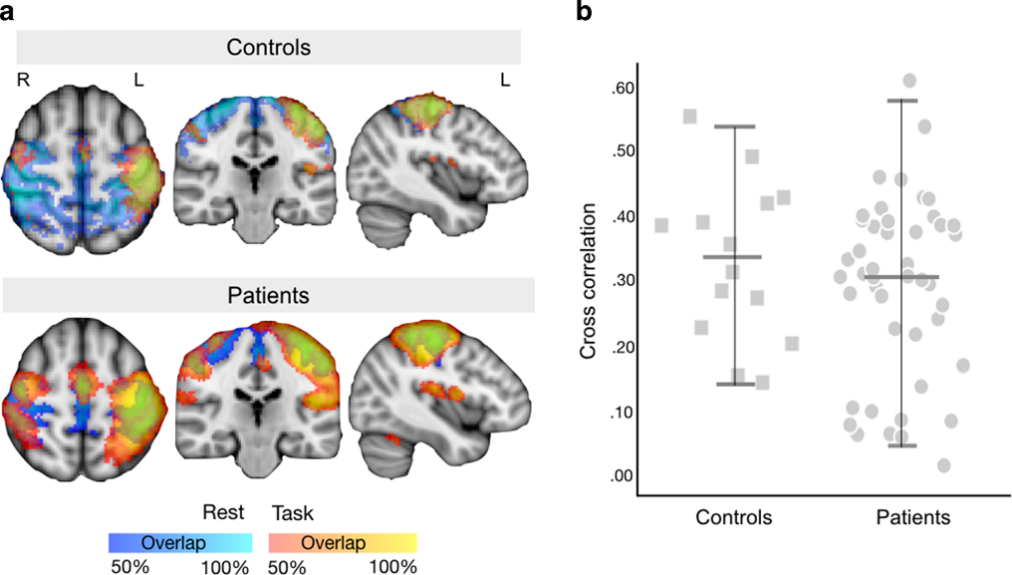
Task and resting fMRI-derived (sensori)motor maps. **a** Overlap heat maps in controls (*n* = 14) and patients (*n* = 45 task, *n* = 71 rest) for task (*red-yellow*) and resting (*blue*) fMRI overlaid onto the template MNI152 brain. Only those voxels shared by at least 50% of each group are shown. **b** Overlap between task and resting maps was computed using spatial cross-correlation. There was no statistical difference in the amount of overlap between task and resting fMRI maps between healthy controls and glioma patients (independent samples t‑test, t = 0.96, *p* = 0.35)

**Fig. 3:**
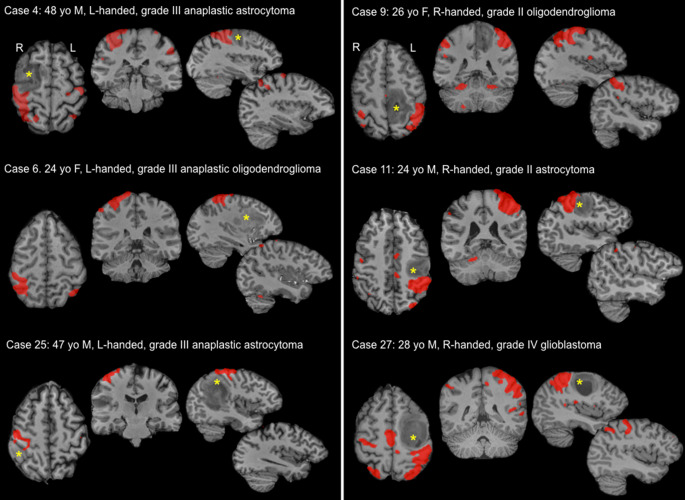
Lack of apparent functional reorganization in individual tfMRI maps. Single subject tfMRI results during finger tapping were visualized to examine evidence for potential functional reorganization in 6 patients with a tumor directly encroaching upon or distorting the peri-Rolandic cortex. All patients had normal muscle power (5/5 on the MRC scale). In each case, the task activation localized to the anatomically expected region on the motor homunculus in the hemisphere contralateral to the hand used to perform the task, and ipsilateral to the tumor. Yellow stars mark the glioma in each case

Task-correlated motor activations were detected in all 14 controls and all 45 (100%) patients undergoing tfMRI. Finger tapping with the dominant hand activated expected regions of the contralateral precentral and postcentral gyri, ipsilateral precentral gyrus and bilateral supplementary motor area (Fig. 2). At the individual level, no evidence for functional reorganization away from the expected sensorimotor cortex was observed (Fig. 3).

For rsfMRI data, DR reconstructed a bilateral network matching the extended sensorimotor network in 14/14 healthy controls (100%) and 64/71 patients (90.1%). In the remaining seven patients, Gaussian gamma mixture modeling and thresholding resulted in few and sparsely distributed voxels (Online Resource Fig. S2). By comparison, visual inspection of individual rsfMRI ICAs identified at least one clear sensorimotor network in all healthy controls and 61/71 patients (85.9%). This difference between DR and visual identification did not reach significance ($\chi$(1) = 1.35, *p* = 0.25). Within healthy controls, both tfMRI and rsfMRI were highly reproducible, but tfMRI was less variable than rsfMRI over time (Online Resource results).

### M1 Correspondence

Task-fMRI activated on average 19% of the M1 mask volume (8785 ± 2477 mm^3^) in controls and 41% (19,108 ± 7390 mm^3^) in patients (t = −7.9, *p* < 0.001). Patients with CS involvement/displacement engaged a larger extent of M1 than patients with tumor remote to the CS (t = −2.4, *p* = 0.020). By comparison, the resting (sensori)motor map included on average 26,932 ± 8003 mm^3^ of the atlas M1 mask (58%) in controls and 21,428 ± 10,656 mm^3^ (46%) in patients (t = 1.8, *p* = 0.07, Online Resource Fig. S3). Patient subgroups (CS affected or not) also did not differ in that respect (t = 1.8, *p* = 0.077). In patients with both task and resting fMRI available (*n* = 45), amount of M1 overlap did not differ between tfMRI and rsfMRI (t = −1.33, *p* = 0.19). Additional Dice similarity metrics are reported in Online Resource results.

### Generalizability of Results

Since tfMRI succeeded in all participants, the impact of temporal acceleration was determined for rsfMRI only. Of the seven patients in whom no resting sensorimotor network was found using DR, four (57.1%) were scanned using basic EPI (TR = 3.5 s) and the other three (42.9%) using intermediate acceleration (TR = 1.56 s). The DR succeeded in all patients with fast acquisition rsfMRI (TR = 0.72 s). Due to the high overall success of DR (90.1%), this difference in template-driven resting network detection did not reach significance ($\chi$(2) = 4.67, *p* = 0.09). The slightly fewer successful visual identifications from single-subject resting ICA reflected differences among scan groups ($\chi$(2) = 17.84, *p* < 0.001). Specifically, sensorimotor networks were detected more frequently in accelerated (*n* = 47/48, 97.9%) than in basic rsfMRI scans (*n* = 14/23, 60.8%). To further explore this finding, one Trio patient was scanned using both a sequence without and with an intermediate acceleration for an equivalent number of volumes (250). Accelerated rsfMRI offered enhanced sensorimotor network detectability in this individual (Online Resource Fig. S4).

**Fig. 4:**
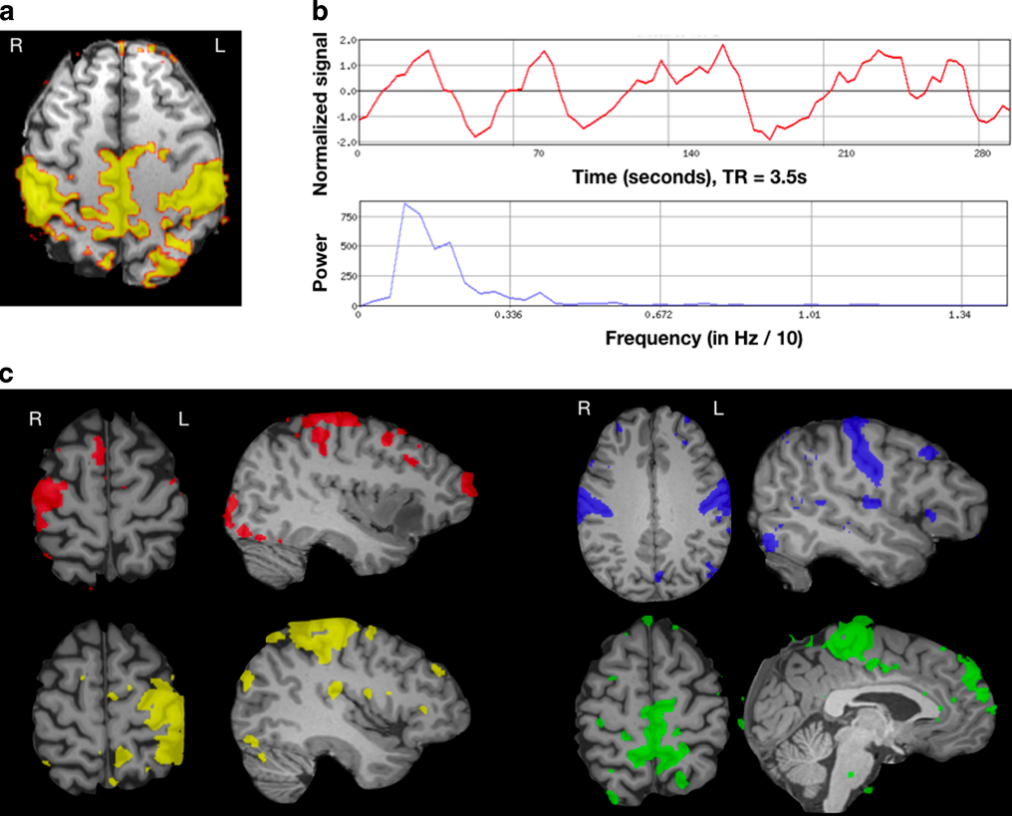
Visual identification of sensorimotor components in individuals’ resting fMRI. Independent Component Analysis (ICA) decomposition of the resting fMRI time series in two representative patients. **a** In some patients (typically with basic clinical rsfMRI acquisitions of low temporal resolution) a single extensive, bilateral sensorimotor network was identified. Visual network identification relied upon 3 criteria: spatial correspondence with the central sulcus (top left), a biologically plausible time-course (top right) and a predominantly low-frequency (0.01–0.05 Hz) power spectrum (bottom right). **b** In another patient, scanned at a high temporal resolution rsfMRI, multiple spatially segregated sensorimotor resting networks were identified, which co-localized with the expected functional divisions of the foot/leg (green), hand/arm (red/yellow; split by hemisphere) and mouth/face (blue) areas along the central sulcus

In terms of subdivisions along the CS, a single extended resting (sensori)motor network was found in 15/61 (24.6%) patients with successful rsfMRI. Spatially segregated sensorimotor networks were identified in the remaining patients. Segregated components localized to the expected face (38/61, 62.3%), hand (38/61, 62.3%) or foot (26/61, 42.6%) motor homunculus subregions (Fig. 4). At least 2 sensorimotor networks were seen in 33 patients (all scanned with temporal acceleration, versus 0/23 patients scanned using a standard sampling rate, $\chi$(2) = 29.6, *p* < 0.001). All 3 functional zones were distinguished in 20 patients, all scanned using temporal acceleration (9/22 Trio, 11/26 Prisma, 0/23 Verio, $\chi$(2) = 13.4, *p* = 0.001). Correspondence to tfMRI available in the same patients varied by sequence acquisition. Patients scanned with temporal acceleration at rest (*n* = 24) showed better correspondence between template-derived sensorimotor and task maps than patients scanned with no acceleration (*n* = 21; t = −2.73, *p* = 0.008). Similarly, spatial concordance between tfMRI and rsfMRI single-subject ICA results was better when selecting the resting map localized to the CS omega sign among accelerated rsfMRI scans (*n* = 17), compared to using the single widespread sensorimotor resting maps identified in basic rsfMRI (*n* = 11; t = −2.68, *p* = 0.013).

### Impact of Tumor Location/Grade

The tfMRI succeeded in all cases, while visual detections from single-subject resting ICA were as frequent when a tumor involved or displaced the CS (28/71, 39.4%) as when it did not (33/71, 46.5%; $\chi$(1) = 0.68, *p* = 0.41). The rsfMRI network detectability did not differ between WHO grades ($\chi$(3) = 2.10, *p* = 0.55).

### Motor Outcomes

Prior to surgery, 5/71 patients (7%) presented with mild or moderate weakness (MRC grading 3–4/5), each with a tumor involving or displacing the peri-Rolandic cortex (5/34, 14.7%; Table 2). Of the patients eight did not undergo resection, four of whom (50%) had a tumor involving the precentral/postcentral region. Reasons for not undergoing resective surgery included refusal of the patient due to associated risks (*n* = 4), rapid tumor progression into multifocal disease (*n* = 2), high seizure activity precluding awake intraoperative stimulation (*n* = 1), and unsuitability for awake surgery (*n* = 1). Among the 63 patients operated on, motor function declined immediately following surgery in 12 (19.1%), including 4/5 (80%) patients with pretreatment weakness. Therefore, new muscle weakness emerged postsurgery in 8/30 patients (26.7%) with tumors affecting or effacing the CS, compared to 0/33 (0%) patients with tumors not affecting the CS ($\chi$(1) = 10.08, *p* = 0.001): 3 months after surgery, seven patients recovered fully, two partially, one had persisting motor weakness, and one patient declined further due to ischemia affecting the corticospinal tract.

The 12 patients with postoperative motor deterioration (Table 4) each had a tumor anatomically involving (*n* = 9) or displacing (*n* = 3) the CS. Among these, rsfMRI failed to detect a sensorimotor network in 4 (33.3%). In 2/4 cases, tfMRI data were available and succeeded when rsfMRI had failed (Online Resource Fig. S5). All 12 patients had undergone surgery with intraoperative mapping/monitoring.

**Table 4:** Surgical outcomes in patients with a glioma affecting or displacing the pre/post-central cortices

Case	Relation to M1	Operated Y/N	Operated awake Y/N	Stimulation errors Y/N	Postoperative deficit Y/N	Resection extent	Recovery Y/N
1	Involving	Y	Y	Y	Y	Near total	Y
2	Involving	Y	Y	Y	Y	Partial	Y
3	Involving	Y	N	–	N	Gross total	–
4	Involving	Y	N	–	N	Subtotal	–
5	Involving	Y	N	–	N	Partial	–
6	Involving	Y	Y	N	N	Gross total	–
7	Involving	Y	Y	Y	Y	Partial	Y
8	Involving	Y	Y	N	N	Near total	–
9	Involving	Y	Y	Y	Y	Near total	Y
10	Involving	Y	Y	Y	Y	Partial	Y
11	Involving	N	–	–	–	–	–
12	Involving	Y	Y	Y	N	Complete	–
13	Involving	N	–	–	–	–	–
14	Involving	Y	Y	Y	N	Near total	–
15	Involving	N	–	–	–	–	–
16	Involving	Y	Y	Y	Y	Gross total	Y
17	Involving	Y	Y	N	N	Complete	–
18	Involving	Y	Y	Y	Y	Biopsy^b^	Partial
19	Involving	N	–	–	–	–	–
20	Involving	Y	Y	N	N	Gross total	–
21	Involving	Y	Y	Y	N	Near total	–
22	Involving	Y	Y	Y	Y	Partial	Y
23	Involving	Y	Y	Y	Y	Partial	N
24	Displacing	Y	N	–	N	Near total	–
25	Displacing	Y	Y	N	N	Subtotal	–
26	Displacing	Y	N	–	N	(CT only)	–
27	Displacing	Y	Y	Y^a^	N	Complete	–
28	Displacing	Y	Y	Y	Y	Near total	Y
29	Displacing	Y	Y	N	N	Complete	–
30	Displacing	Y	N	–	N	Subtotal	–
31	Displacing	Y	N	–	N	Biopsy	–
32	Displacing	Y	N	–	Y	Partial	Y
33	Displacing	Y	Y	Y	N	Partial	–
34	Displacing	Y	Y	Y	Y	Partial	N

^a^Negative cortical stimulation but positive subcortical motor stimulation sites

^b^Only a biopsy could be performed due to immediate leg weakness on biopsy. Intraoperative stimulation errors were defined as a loss of muscle power/range during continuous movements, or involuntary movements/sensations during rest elicited by the application of an electrical current using a bipolar cortical stimulator. Cortical stimulation was performed using an Ojemann OCS2 (Integra LifeSciences Co, Saint Priest, France [23]) or Nicolet (Natus Neuro Incorporated, Middleton, WI, USA [24]) stimulator delivering a biphasic current (200 μs pulse duration, 60 Hz pulse frequency, amplitude range between 1–6 mA)

## Discussion

This study investigated the success of tfMRI, rsfMRI and expert radiological review to delineate the sensorimotor cortex in glioma patients. Anatomical review of high-resolution 3D T1-weighted images unequivocally located the ipsilesional CS in 98.6% of patients. Comparatively, motor tfMRI succeeded in 100%, while rsfMRI failed to localize a sensorimotor network in up to 14.1% of patients. Specifically, rsfMRI failed in four patients who experienced postoperative deterioration that was anticipated by anatomical location and/or tfMRI. Variability of rsfMRI was not influenced by tumor location or grade but instead by temporal sampling rate. When expert neuroradiological judgment diverged (1.4% of cases), tfMRI successfully localized the primary hand motor cortex. Clinically, surgical risks to motor function are therefore largely predicted by anatomical criteria. Only in cases where CS anatomy is severely obscured by proximate pathology, motor tfMRI, over and above standard rsfMRI, may support localization of M1.

Natural variability can hinder accurate identification of the CS; however, 3D MRI techniques identify up to 10 anatomical landmarks [25] that robustly localize the CS. In this series, neuroradiological review identified the CS in 70 of 71 patients, corresponding to 33 of 34 (97.1%) gliomas directly infiltrating or distorting the peri-Rolandic cortices. Only one patient (2.9%) showed sulcal effacement sufficient to cast doubt on CS location. These results are consistent with the large series of Yousry et al. [13], who reported failed CS identification in only 4/198 patients (2%) due to mass effects. Motor functions, however, are variably localized [2] and can decouple from the underlying anatomy [2, 26–28]. Potential reorganization of motor functions motivates tfMRI acquisitions even when radiological CS delineation is preserved; however, while the patients with a tumor involving/displacing the CS activated a wider extent of M1, there was no indication for overt functional reorganization at the individual level.

Frequently cited limitations of tfMRI are failures due to variable compliance, pre-existing deficits [29, 30] and pathological confounds [31]. In previous direct comparisons, rsfMRI showed sensorimotor network localization comparable [32] or superior [10] to tfMRI. The lower success of rsfMRI in the present series seems, therefore, at first surprising; however, the former study used a faster TR for rest (2 s) than task (3 s) while the latter compared 4 min of tfMRI to 15 min of rsfMRI. A third study with matched sequences involved only four patients [11]. Temporal acceleration is reported to increase statistical power in rsfMRI [33, 34]. Concordantly, temporal resolution was a significant determinant of resting sensorimotor network detectability in the present series of patients: detection failed in nine cases scanned using an unaccelerated clinical acquisition but not in any of the patients scanned using fast acceleration. Others [35] previously determined that short (<10 min approximately) basic acquisition scans (TR between 2.16 s and 3 s) reduced the accuracy of RSN detection, likely due to low signal to noise or temporal fluctuations between RSNs. The present results corroborate this finding in the clinical setting and further substantiate that tfMRI activations elicited by short targeted behaviors generate more robust, clinically informative regional responses for functional localization than unaccelerated rsfMRI acquisitions [36]. Of course, it remains possible that rsfMRI would out-perform tfMRI in patients with greater sensorimotor deficits than were present in this cohort.

The primary clinical application of fMRI is to predict surgical risks and, accordingly, outcomes. Proximity of tfMRI sensorimotor activations (and essential corticospinal fiber tracts [37]) to tumor margins, while problematic as a statistically-dependent measure, is informative to predict postsurgical deficits [38–40]. However, among 12 patients in this series who experienced new or exacerbated motor deficits following surgery, tfMRI largely confirmed anatomical information, i.e. that surgery proximal to the CS was associated with a high risk of motor deterioration. In nine patients, the tumor involved functionally active, anatomically identified peri-Rolandic cortex. In the remaining three, motor mapping by tfMRI was not directly inform sensorimotor outcomes. Instead, transient deficits were attributed to SMA (supplementary motor area) syndrome and postsurgical edema in two patients. Permanent morbidity in one patient resulted from ischemia affecting the corticospinal tract. Clinical decisions were aided by tfMRI in only one patient, who underwent a biopsy because the risks of resective surgery were not acceptable for the patient.

This study focused on the sensorimotor network which generally shows tight anatomicofunctional coupling (i.e. absolute cortical representation [41]). Thereby, it was possible to evaluate the comparative accuracy of task versus resting fMRI for the localization of sensorimotor functions. The sensorimotor network was chosen since, in our experience, motor mapping remains among the, if not the most commonly requested presurgical fMRI applications. The data confirm that when expert neuroradiological review is available, sensorimotor fMRI mapping will, in most cases, become superfluous. Note that this conclusion does not extend to other functional systems, such as language, which cannot be reliably identified using anatomical landmarks alone. The potential added value of rsfMRI will be much more difficult to establish for language mapping due to the heterogenous representation of different language networks in the brain. However, rsfMRI for language mapping showed some initial promise [42] and warrants further research.

Additionally, by analyzing all patients with available imaging data at two sites, potential selection biases based on tumor pathological grade, surgical selection or motor performance outcome were minimized and the likely generalizability of the findings to other glioma populations were maximized. However, a limitation of the study is that a single task was used to compare tfMRI with rsfMRI, whereas multiple tasks are needed to localize discrete functional areas along the motor homunculus. A prospective study using multiple, clinically relevant motor tasks (probing, for example, leg, hand and tongue functions), and repeating resting fMRI using multiple sequence acceleration factors within patients should be conducted to confirm the findings. Furthermore, ICA was used to match tfMRI and rsfMRI analyses. Alternative seed-based analysis, however, depends on accurate a priori identification of functional regions of interest.

In conclusion, M1 can be anatomically identified for most gliomas despite mass effect, infiltration, perifocal edema and sulcal effacement. In ambiguous cases without substantial hemiparesis who can perform the task, tfMRI is effective and robust to delineate hand motor functions. The use of rsfMRI may offer advantages when tfMRI is not feasible but the data indicate that it requires rapid (or prohibitively long) sampling to attain similar statistical sensitivity.

## Caption Electronic Supplementary Material

Supplementary methods and results, including additional reproducibility and task-resting FMRI overlap results with corresponding data Tables and Figures. Additional supplementary Figures depicting the fMRI task, resting fMRI dual regression analysis failures, a case illustration of the impact of accelerated acquisition sequences and FMRI results in patients with post-operative motor deterioration.
