# Supplementary material for: Presurgical Localization of the Primary Sensorimotor Cortex in Gliomas: When is Resting State FMRI Beneficial and Sufficient?
Source: Clin Neuroradiol. 2020 Apr 9;31(1):245–56. doi: 10.1007/s00062-020-00879-1 (PMC7943510; doi:10.1007/s00062-020-00879-1)
Supplement: Supplementary file 1 — Supplementary methods and results, including additional reproducibility and task-resting FMRI overlap results with corresponding data Tables and Figures. Additional supplementary Figures depicting the fMRI task, resting fMRI dual regression analysis failures, a case illustration of the impact of accelerated acquisition sequences and FMRI results in patients with post-operative motor deterioration. [file 62_2020_879_MOESM1_ESM.docx]

**Supplementary Materials and Results accompanying the article “Pre-surgical localization of the primary sensorimotor cortex in gliomas: When is resting-state FMRI beneficial and sufficient?”**

***Methods***

*Reproducibility analyses*

Task and resting FMRI scans were repeated twice in 14 healthy volunteers, 6 months apart. One volunteer completed just a single task run. Task (n=13) and resting (n=14) FMRI reproducibility between the two visits was quantified in two ways: Firstly, we computed the spatial cross-correlation between the tFMRI-associated thresholded z-statistical maps derived from visit 1 and visit 2, and between the rsfMRI thresholded z-statistical sensorimotor network maps identified using dual regression and acquired at each visit. A cross-correlation of 1 would represent a perfect correlation between the two maps. Secondly, we performed non-parametric permutation testing to directly compare the task and resting FMRI maps obtained at each visit. The unthresholded z-statistic sensorimotor maps were submitted to a one-sample t-test using non-parametric permutation testing (using 5000 permutations to provide p<0.05, family-wise error corrected). For this analysis, a difference map was calculated by subtracting the visit 2 map from the respective visit 1 map of each participant in order to identify any voxels showing a linear trend over time (i.e., a positive or negative deviation from zero between the two time-points). Permutation testing was performed using ‘randomise’ (part of FSL; [1]).

*Resting FMRI dual regression (DR) analysis*

Spatial maps generated by independent component analysis (ICA) decomposition each have a characteristic time-course. Therefore, the first regression step searched for a match between the temporal characteristics of each participant’s rsFMRI data and the set of 10 consistently identified template resting state networks [2]. Next, in the second step of DR, voxels were identified in each person’s data that shared this time-course, resulting in individual-level spatial maps matching each of the 10 template networks. The sensorimotor network was selected out from among the 10 networks and statistical inference performed on each participant’s resulting sensorimotor network using Gaussian Gamma Mixture Model and alternative hypothesis testing (p>0.5, testing each voxel’s probability of being ‘active’ vs ‘noise’ [3]).

*Histologically-defined primary motor cortex (M1) mask*

To compute spatial overlap between anatomical definitions of the central sulcus (CS) and FMRI-derived (sensori)motor maps, we selected a template anatomical “motor cortex” mask. From the Jülich histological atlas [4], the anatomical labels corresponding to the probabilistic location of the anterior and posterior primary motor cortex (M1, Brodmann cytoarchitectonic area 4) were identified in the left and right hemispheres. The labels were thresholded to retain voxels histologically corresponding to M1 in at least 33% of the atlas population, binarized and summed to create a bilateral M1 mask. The resulting atlas mask was registered to each participant’s T1-weighted anatomical scan using nonlinear registration by FNIRT (part of FSL). Registration of the template histological mask was verified in every participant by inspecting how well the CS aligned. Due to intractable poor registration, 1 / 71 (1%) patient was excluded from this analysis. In the remaining participants, the task and rsFMRI (sensori)motor statistical maps were converted into binary maps to determine the within-subject Dice similarity coefficient between the respective FMRI maps and the anatomical motor cortex.

Because spatial extents of activation in FMRI are highly dependent on statistical thresholding, we quantified the Dice index for binary maps generated by iteratively thresholding the z-statistical maps at a proportion of the maximum activation for each individual. The maximum z-statistic range was determined for each individual’s resting and task-derived (sensori)motor maps. These z-statistical maps were then thresholded at 10%, 25%, 50% and 75% of the maximum range and converted into binary masks to calculate the respective Dice coefficients. The maximum Dice coefficient is 1, representing a perfect match between two binary images. Dice similarly indexes were calculated in Matlab (Mathworks, R2016a).

***Results***

*Reproducibility results*

Test-retest comparisons among healthy controls showed high reproducibility of task- and resting-FMRI spatial maps repeated 6 months apart (tFMRI cross-correlation: mean 0.76±0.11; rsFMRI cross-correlation: mean 0.61±0.17). Additionally, there was no significant difference in voxel-wise activations between the two visits in either task (nonparametric one sample t-test, p=0.14) or resting FMRI maps (p=0.33) (Fig. S6). Between-session reproducibility (cross-correlation) was greater for task than for resting FMRI (t=3.87, p=0.002).

*Task vs resting FMRI spatial overlap*

Both tFMRI and rsFMRI were available in 14 controls and 45 patients. Task and resting FMRI (sensori)motor maps showed moderate-to-low overlap, with high variability in the amount of correspondence among individuals. Average cross-correlation metrics were similar between healthy controls (mean 0.32±0.17), and patients (mean 0.28±0.13) with no significant difference in the amount of task vs resting FMRI overlap between patients and controls (t=0.89, p=0.38).

*FMRI overlap with M1*

Dice coefficient estimates of overlap were sensitive to the level of statistical thresholding applied to the activation maps (Fig. S3). The highest index of similarity was measured at a level of statistical thresholding representing 10% of each individual’s maximum z-statistical activation range. Patients showed similar amounts of task overlap with the anatomical M1 mask compared to healthy controls (t=0.87, p=0.39; patients mean Dice 0.12±0.04 vs controls mean Dice 0.13±0.03).

Comparing amount of overlap between the DR-derived sensorimotor RSN map and the anatomical atlas M1 mask (Fig. S3), there was no difference between patients (mean Dice index: 0.17±0.08) and controls (mean Dice index: 0.20±0.08; t=1.36, p=0.18). However, similarity with the anatomical M1 was higher for rsFMRI-derived than for tFMRI sensorimotor maps in patients (t=-4.77, p<0.001).

**Table S1. Test-retest results in healthy controls**

|  | Motor task FMRI (visit 1 - visit 2) | Resting FMRI (visit 1 - visit 2) |
| --- | --- | --- |
| Participant | Cross-correlation | Cross-correlation |
| 1 (417) | 0.84 | 0.42 |
| 2 (423) | 0.87 | 0.76 |
| 3 (425) | 0.85 | 0.74 |
| 4 (428) | 0.61 | 0.44 |
| 5 (429) | - | 0.41 |
| 6 (430) | 0.79 | 0.61 |
| 7 (449) | 0.82 | 0.74 |
| 8 (453) | 0.71 | 0.70 |
| 9 (454) | 0.82 | 0.74 |
| 10 (455) | 0.83 | 0.72 |
| 11 (458) | 0.82 | 0.85 |
| 12 (459) | 0.49 | 0.31 |
| 13 (461) | 0.66 | 0.70 |
| 14 (462) | 0.80 | 0.42 |
| ***Mean*** | ***0.76 ± 0.11*** | ***0.61 ± 0.17*** |

*Table S1 Legend*. Cross-correlations between data acquired 6 months apart in healthy volunteers for task FMRI (n=13) and resting FMRI (n=14). Cross-correlations were computed between the Gaussian Gamma Mixture Model thresholded statistical (sensori)motor map derived from each visit.

**Supplementary Fig S1. FMRI adaptation of the Corsi block tapping task**


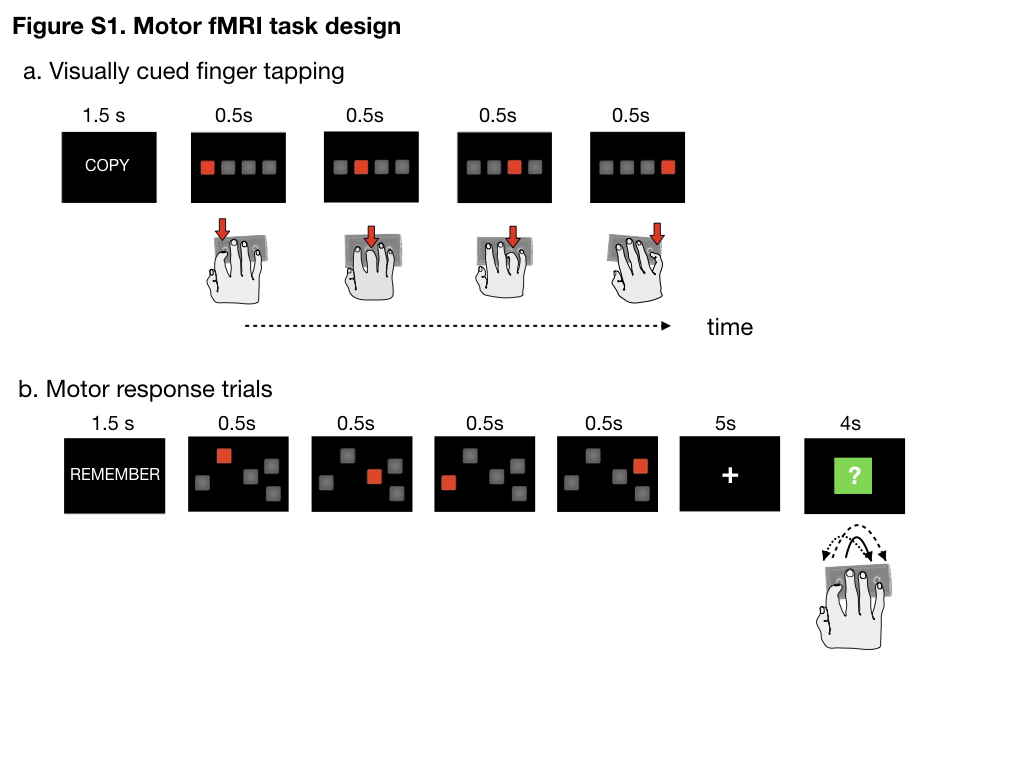


*Fig S1 Legend***.** FMRI adaptation of the classical Corsi block tapping task [5]. Participants performed 5 repetitions of visually paced (0.5Hz) sequential finger tapping using a dedicated MRI compatible button box at the start and end of the task. During the remainder of the task, participants were visually presented with sequences of 4 squares, presented one after the other in a unique location on the screen. Participants were asked to remember the sequence and, after a short delay (5s), to replicate the remembered order by pressing the 4 respective buttons on the button box. These response trials were added to the sequential motor trials to provide a total of 112 finger presses, corresponding to a total duration of 56s within a total overall task time of 07:30.

**Supplementary Fig S2. Resting FMRI dual regression failures.**


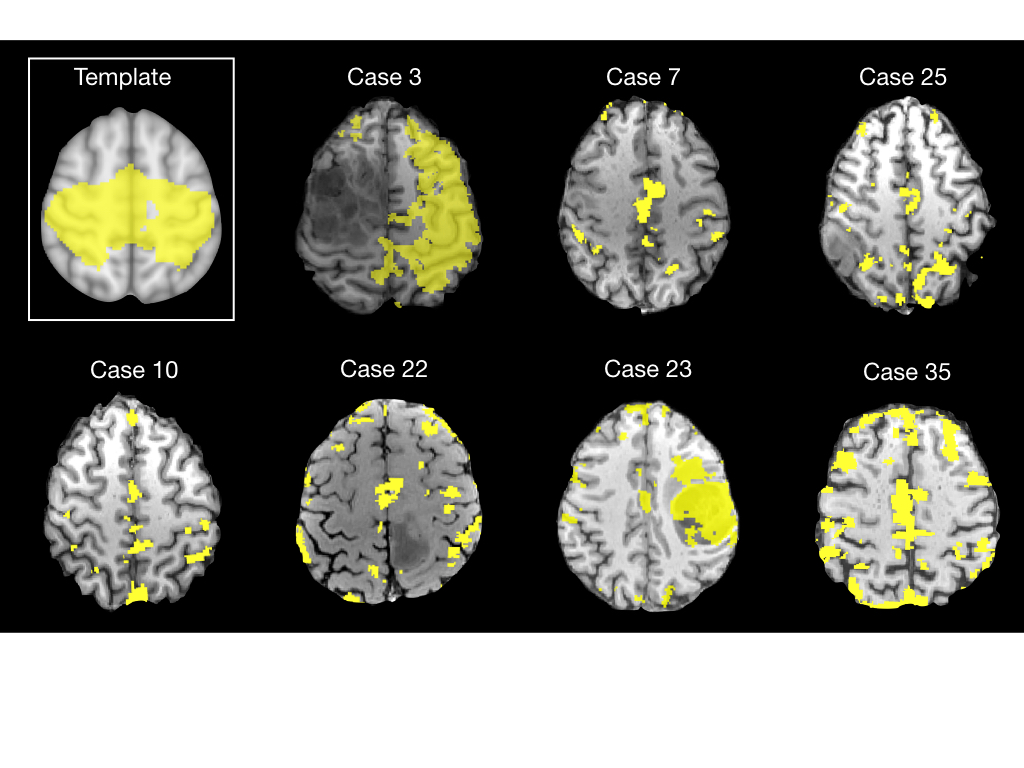


*Fig S2 Legend*. Dual Regression was used to identify the bilateral sensorimotor network corresponding to the template from the well-validated set of 10 resting state networks [2] in every participant’s resting FMRI data. DR succeeded in all 14 healthy controls and in 64 of the 71 patients. In the 7 remaining patients, the thresholded z-statistic map resulting from dual regression did not correspond to a clearly localized sensorimotor network.

**Supplementary Fig S3. Overlap of FMRI (sensori)motor maps with histologically-predicted M1.**


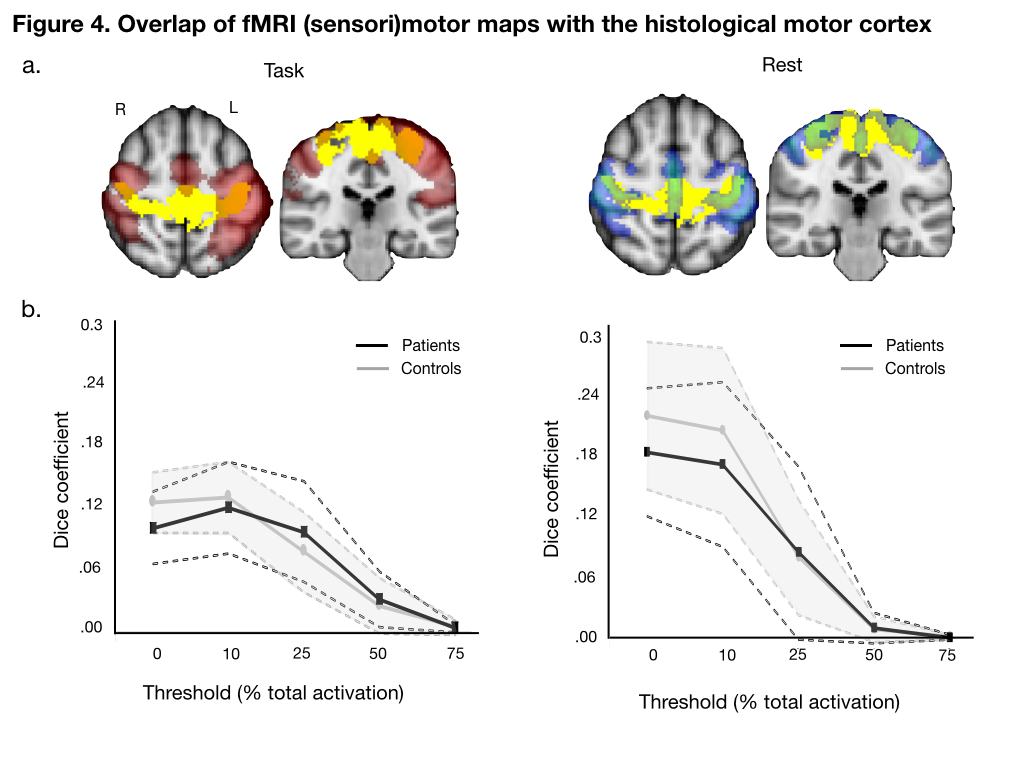


*Fig S3 Legend*. A. Group average task FMRI (red) and resting FMRI (blue) sensorimotor networks generally co-localized to the histologically-predicted primary motor cortex (M1, yellow; overlap with task FMRI in orange, with resting FMRI in green). As expected, the task-derived sensorimotor map evoked by unilateral finger tapping was more lateralized than the network derived from rest. B. Quantifying the extent of spatial overlap by Dice coefficients revealed a sensitivity of overlap measures to map thresholding. At low (liberal) levels of thresholding (0 and 10%), resting FMRI sensorimotor maps in patients engaged a larger extent of M1 than the network derived from task FMRI (paired samples t-tests, p<0.001 at both threshold levels).

**Supplementary Fig S4. Sensorimotor network detection in a patient scanned with both basic and accelerated resting FMRI sequences.**

**
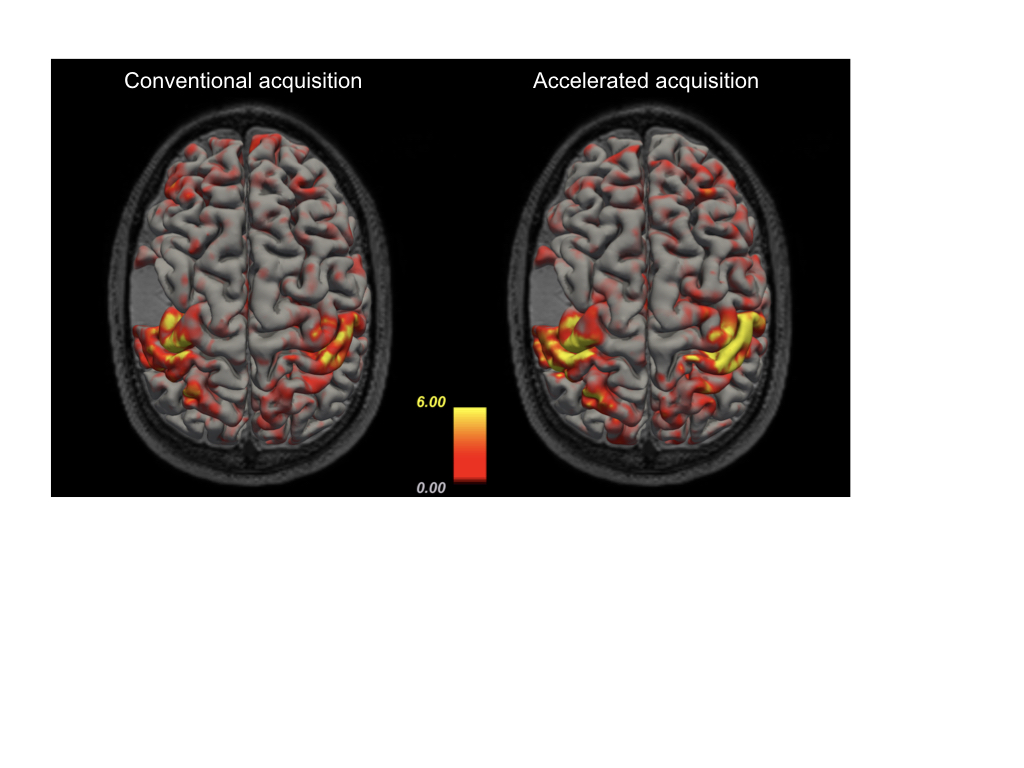
**

*Fig S4 Legend*. Improved (sensori)motor mapping by whole-brain resting FMRI of intermediate acceleration as opposed to an unaccelerated acquisition in a patient with a left precentral low-grade glioma prior to resective surgery. The patient suffered from partial focal mouth seizures involving both eyebrows (bilateral cranial nerve VII innervation), the contralateral mouth corner (contralateral N. VII innervation), chewing, vocalizations and speech arrest (N. V, IX, X and XII innervation), corresponding to the motor homunculus representation below the handknob. Resting-state FMRI reveals statistical gains (color-coded Z-statistic maps from dual regression) of rapid temporal sampling over conventional resting FMRI without temporal acceleration, despite the shorter acquisition time (both 250 volumes, corresponding to 6.5 vs 14 minutes).

**Supplementary Fig S5. Anatomical and FMRI localization of the motor cortex in patients with post-operative motor declines**


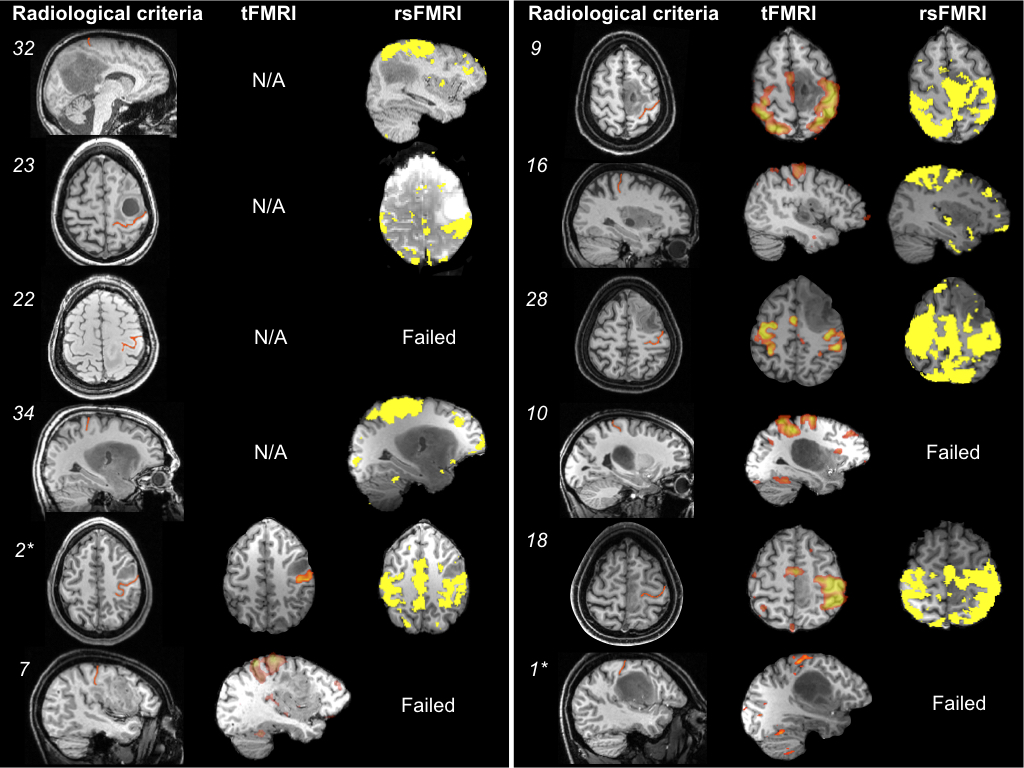


*Fig S5 Legend*. The central sulcus (red line, anatomy column) was identified by anatomical landmarks in all 12 patients who experienced a post-surgical deterioration in motor performance (new onset or deteriorated contralateral hemiparesis). In 4 of these cases, resting FMRI failed to identify a sensorimotor network (3 scanned at low temporal resolution and 1 with an intermediate acceleration). Task FMRI data were acquired in 8 of these cases performing finger tapping, or a separate additional lip pursing task when the tumor was located very laterally along the central sulcus (cases 1 and 2, marked with an asterisk). Task FMRI successfully localized motor functions in 3 / 3 patients in whom it was acquired and in whom resting FMRI had failed.

**Supplementary Figure S6. Test-retest reproducibility in healthy controls**


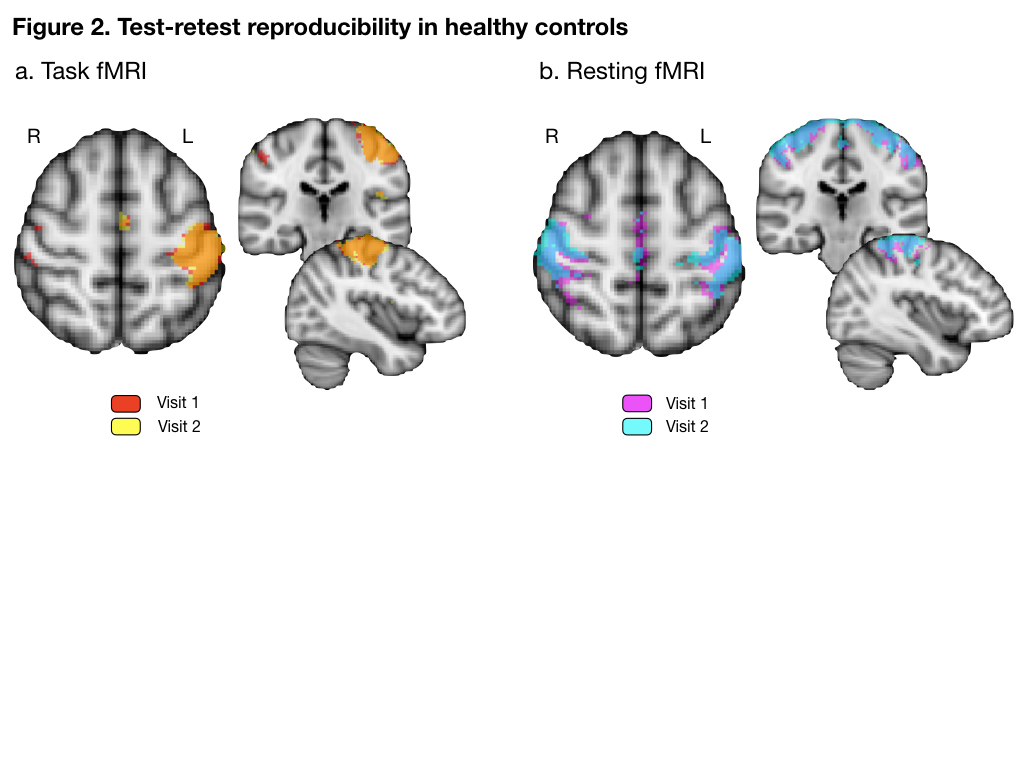


*Fig S6 Legend. a.* Heat maps showing the task-associated sensorimotor maps for all controls derived from visit 1 (red) and visit 2 (yellow; overlap in orange), depicted on the MNI152 template brain. The spatial location of finger-tapping task-related activation was highly reproducible across two visits, acquired 6 months apart. No significant difference was found in the z-statistical maps between the two task visits (nonparametric one same t-test, p=0.14, 5000 permutations). *b.* Similar overlap maps depicting the resting sensorimotor maps derived from visit 1 (magenta) and visit 2 (light blue; overlap in blue), again revealing high correspondence within healthy subjects over time, with no difference in the z-score maps between visits (permutation test, p=0.33).

**Supplementary Figure S7. Anatomical variants of the central sulcus**


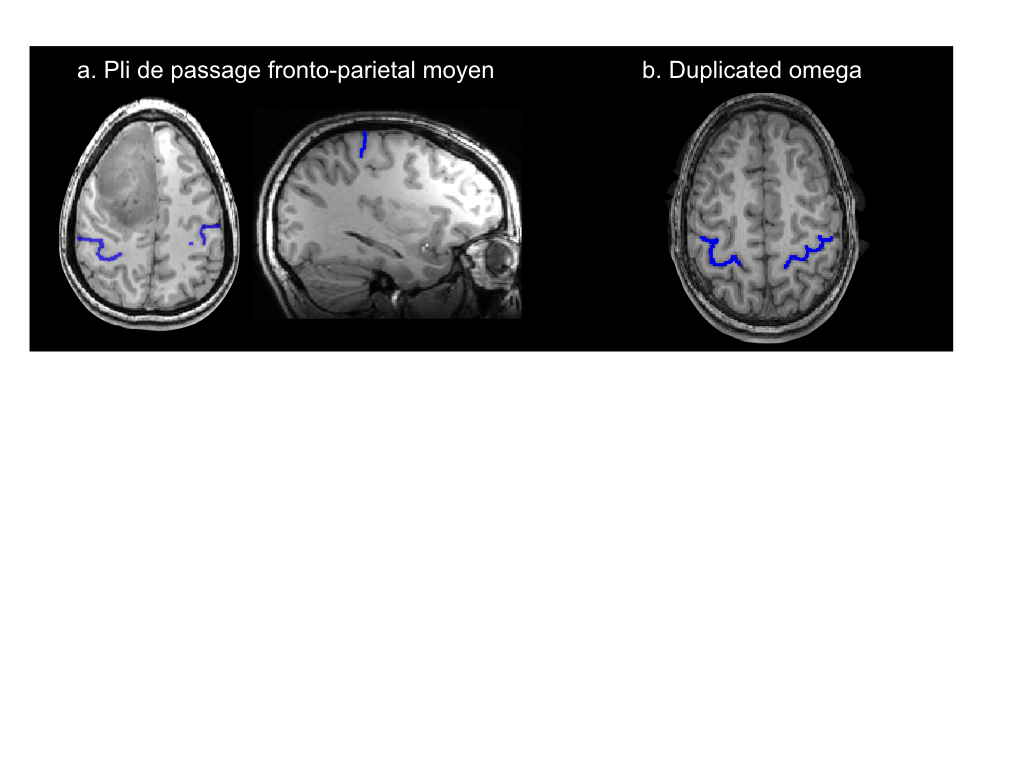


*Fig S7 Legend.* Two variants of the central sulcus anatomy. a. Illustration of a rare incomplete “pli de passage fronto-parietal moyen” [6] connecting the pre- and post-central gyri in the left hemisphere. b. duplication of the inverted ‘omega’ sign in a left-handed individual.

**Supplementary References**

1. Winkler AM, Ridgway GR, Webster MA, Smith SM, Nichols TE. Permutation inference for the general linear model. NeuroImage. 2014;92:381-97. doi:10.1016/j.neuroimage.2014.01.060.

2. Smith SM, Fox PT, Miller KL, Glahn DC, Fox PM, Mackay CE et al. Correspondence of the brain's functional architecture during activation and rest. Proceedings of the National Academy of Sciences of the United States of America. 2009;106(31):13040-5. doi:10.1073/pnas.0905267106.

3. Beckmann CF, DeLuca M, Devlin JT, Smith SM. Investigations into resting-state connectivity using independent component analysis. Philosophical transactions of the Royal Society of London Series B, Biological sciences. 2005;360(1457):1001-13. doi:10.1098/rstb.2005.1634.

4. Geyer S, Ledberg A, Schleicher A, Kinomura S, Schormann T, Burgel U et al. Two different areas within the primary motor cortex of man. Nature. 1996;382(6594):805-7. doi:10.1038/382805a0.

5. Corsi PM. Human memory and the medial temporal region of the brain. . Dis. Abstr. Intl. 34, 891B: McGill; 1972.

6. Alkadhi H, Kollias SS. Pli de passage fronto-parietal moyen of broca separates the motor homunculus. AJNR American journal of neuroradiology. 2004;25(5):809-12.
